# Johnson County Medical Society

**Published:** 1896-04

**Authors:** W. E. Menefee

**Affiliations:** Sec’y Johnson Co. Med. Society; Cleburne, Texas


### Johnson County Medical Society.

CLEBURNE, TEXAS, April 4, 1896.

*Editors Texas Medical Journal:*

Will you please publish the following resolution, passed by Johnson County Medical Society at the last meeting:

*Resolved*, That we learn with pleasure the interest being taken in the State Medical Association and the unanimity of the physicians of the State in upholding the same. We send greeting to our worthy president and bid him God speed in his work.

W. E. MENEFEE, M. D.,
Sec'y Johnson Co. Med. Society.

